# Transcriptome analysis of activated charcoal-induced growth promotion of wheat seedlings in tissue culture

**DOI:** 10.1186/s12863-020-00877-9

**Published:** 2020-07-06

**Authors:** Fu-shuang Dong, Meng-yu lv, Jin-ping Wang, Xue-ping Shi, Xin-xia Liang, Yong-wei Liu, Fan Yang, He Zhao, Jian-Fang Chai, Shuo Zhou

**Affiliations:** 1 Institute of Genetics and Physiology, Hebei Academy of Agriculture and Forestry Sciences, Plant Genetic Transformation Center of Hebei Province, Shijiazhuang, 050000 China; 2 Institute of Millet Crops, Hebei Academy of Agriculture and Forestry Sciences, Shijiazhuang, 050000 China; 3 The Semi-arid Agriculture Engineering & Technology Research Center of P.R. China, Shijiazhuang, 050000 China

**Keywords:** RNA sequencing, Wheat, Immature embryo culture, Phenylpropanoid biosynthesis, Plant hormone signaling

## Abstract

**Background:**

Activated charcoal (AC) is highly adsorbent and is often used to promote seedling growth in plant tissue culture; however, the underlying molecular mechanism remains unclear. In this study, root and leaf tissues of 10-day-old seedlings grown via immature embryo culture in the presence or absence of AC in the culture medium were subjected to global transcriptome analysis by RNA sequencing to provide insights into the effects of AC on seedling growth.

**Results:**

In total, we identified 18,555 differentially expressed genes (DEGs). Of these, 11,182 were detected in the roots and 7373 in the leaves. In seedlings grown in the presence of AC, 9460 DEGs were upregulated and 7483 DEGs were downregulated in the presence of AC as compared to the control. Kyoto Encyclopedia of Genes and Genomes (KEGG) pathway analysis revealed 254 DEG-enriched pathways, 226 of which were common between roots and leaves. Further analysis of the major metabolic pathways revealed that AC stimulated the expression of nine genes in the phenylpropanoid biosynthesis pathway, including *PLA, CYP73A, COMT, CYP84A,* and *4CL*, the protein products of which promote cell differentiation and seedling growth. Further, AC upregulated genes involved in plant hormone signaling related to stress resistance and disease resistance, including *EIN3, BZR1, JAR1, JAZ,* and *PR1*, and downregulated genes related to plant growth inhibition, including *BKI1, ARR-B, DELLA,* and *ABF*.

**Conclusions:**

Growth medium containing AC promotes seedling growth by increasing the expression of certain genes in the phenylpropanoid biosynthesis pathway, which are related to cell differentiation and seedling growth, as well as genes involved in plant hormone signaling, which is related to resistance.

## Background

Bread wheat (*Triticum aestivum* L.) is the most widely grown crop globally, with a cropping area of more than 220 million hectares. It is the staple food for 30% of the global population. However, wheat yields have been affected by global climate change, and new resistant varieties are urgently needed, which is a challenge to be addressed through wheat breeding. As a conventional technique, embryo culture has been widely used in distant hybridization, rapid crop development, and haploid breeding and has promoted the development of new wheat breeds.

Activated charcoal (AC) is a porous carbonized substance with a large inner surface area on which many substances can be adsorbed. AC is often used in plant tissue culture to improve growth and development [1]. It can adsorb harmful substances present in culture media, including impurities in agar, 5-hydroxymethylfurfural produced by sucrose during high-pressure sterilization, and phenoquinones secreted by explants during culture, as well as beneficial substances available in culture media, such as growth regulators, vitamin B6, folic acid, and nicotinic acid [2]. There are many reports on its effects such as anti-browning, improvement of primary culture survival rates, promotion of bud proliferation and seedling growth in the dark, and promotion of rooting [3–7]. However, the mechanism of action of AC in promoting plant growth has been rarely reported.

In recent years, high-throughput sequencing technologies have been widely used in plant research, and their efficiency has dramatically improved [8–11]. In this study, gene expression in 10-day-old wheat seedlings cultured in the presence or absence of AC was compared through transcriptome sequencing. With this study, we aimed to lay a foundation for further study of the mechanisms by which AC promotes the development of immature wheat embryos. Genes that promote wheat growth were thoroughly analyzed to provide a theoretical basis for breeding high-yield wheat varieties.

## Results

### Effect of AC on physiological and biochemical indices of wheat seedlings

**Fig. 1:**
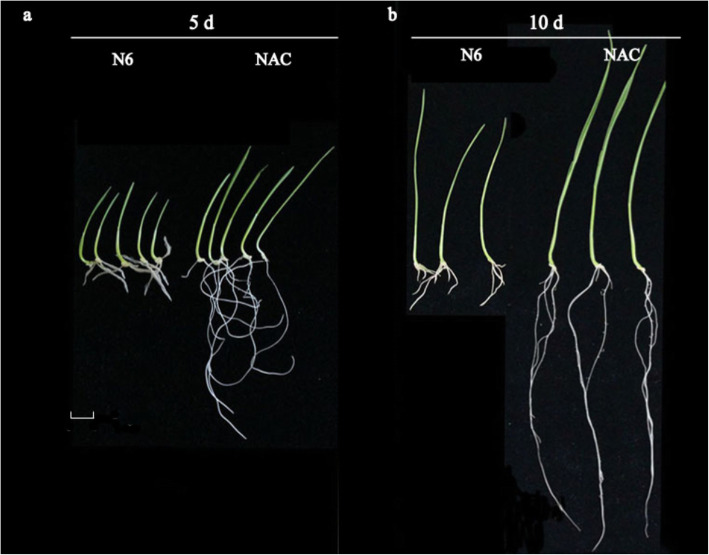
Images showing wheat seedlings grown for 5 days (**a**) or 10 days (**b**) in NAC (basal medium supplemented with 4 g/LAC) or N6 (basal medium). Bar = 1 cm

**Fig. 2:**
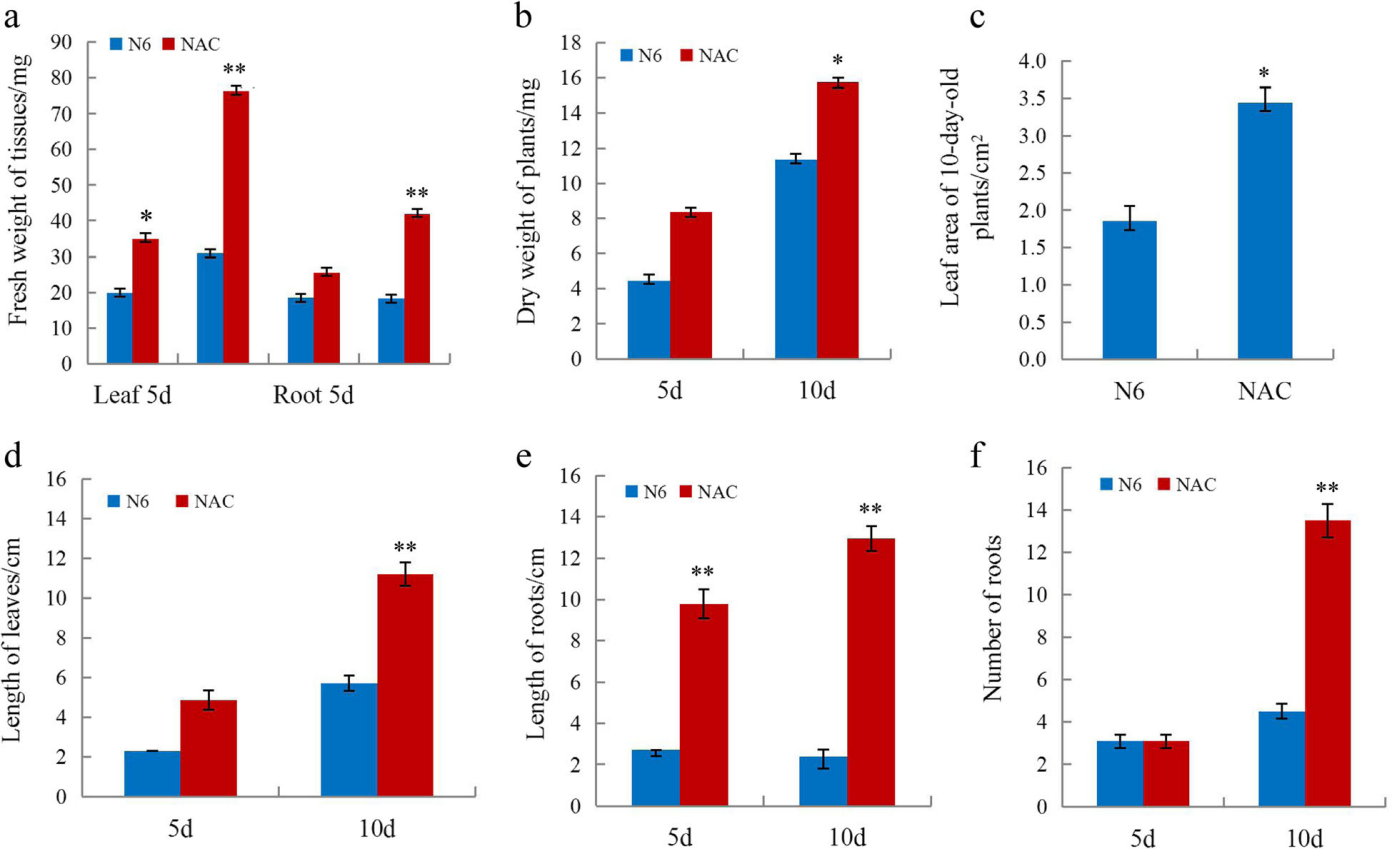
Comparison of growth indices for 5- and 10-day-old seedlings grown on NAC or N6. Data are shown separately for leaves and roots. **a** Fresh seedling weight (leaves plus roots). **b** Dry weight. **c** First-leaf area for 10-day-old seedlings. **d** Leaf length. **e** Root length. **f** Root number. *P < 0.05, ** *P* < 0.01, *t-*test

**Fig. 3:**
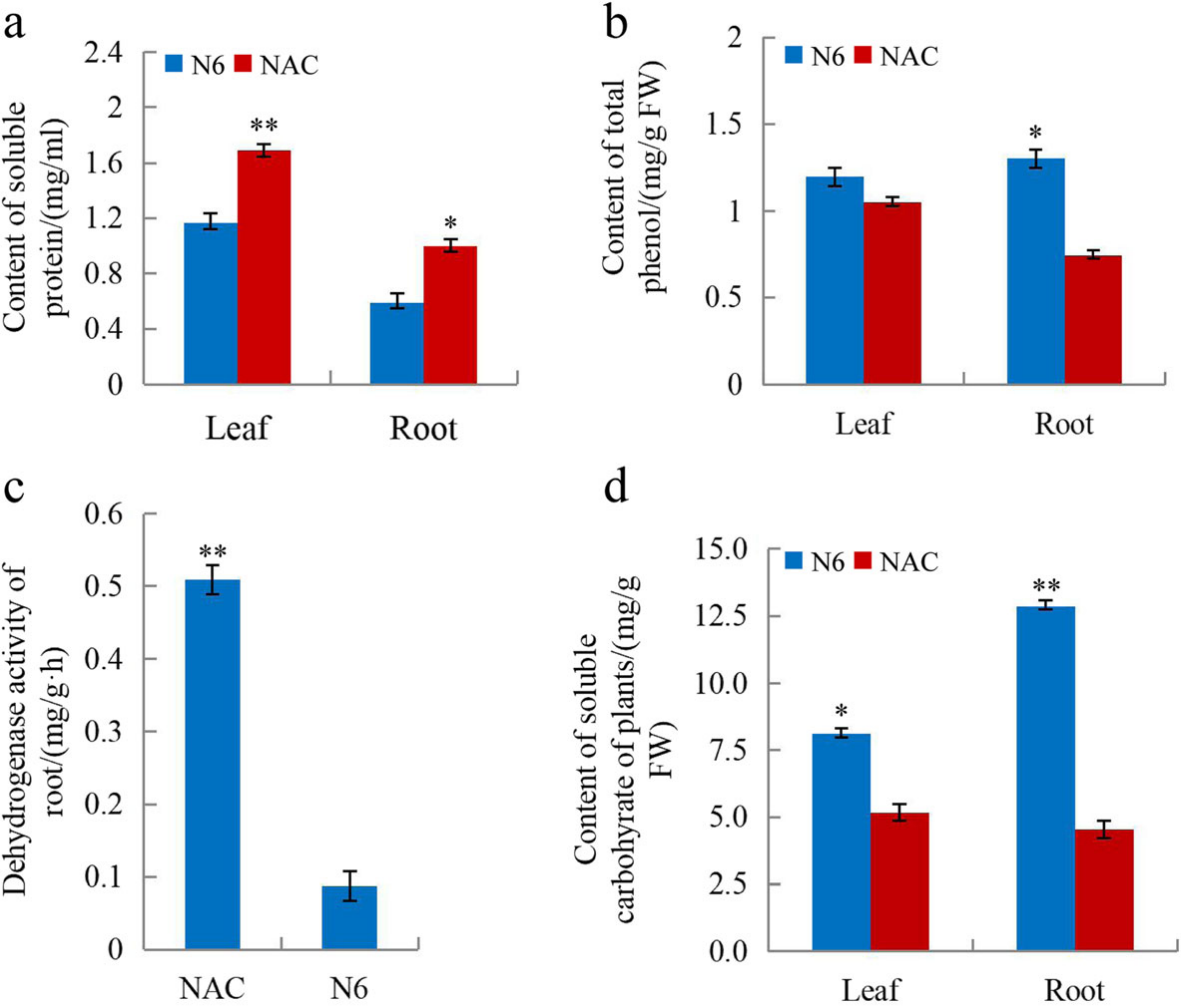
Comparison of biochemical indices for 10-day-old seedlings grown on NAC or N6. Data are shown separately for leaves and roots. **a** Soluble protein content. **b** Total phenol content. **c** Root dehydrogenase activity. **d** Soluble carbohydrate content in the seedlings (mg/g FW). *P < 0.05, **P < 0.01, *t*-test

Briefly, we grew seedlings from scutella in base medium (N6 supplemented with 0.02 mg/L NAA and 0.05 mg/L 6-BA) or NAC (base medium supplemented with 4 g/L AC) in vitro, and seedlings were collected after 5 and/or 10 days for physiological and biochemical analyses as described below in the Methods section. The leaf area was not determined in 5-day-old seedlings, as the leaves are not unfolded at this stage. For 5- as well as 10-day-old seedlings, the growth rate was significantly higher (*P* < 0.05) on NAC than on base medium (Figs. 1 and 2). The results of biochemical analyses of 10-day-old seedlings revealed that NAC promoted root activity and significantly increased the soluble protein content in wheat seedlings compared to N6 medium, whereas the total phenol and soluble sugar contents were lower than on N6 medium (Fig. 3) (*P* < 0.05).

### RNA sequencing analysis of seedlings grown on N6 and NAC

**Fig. 4:**
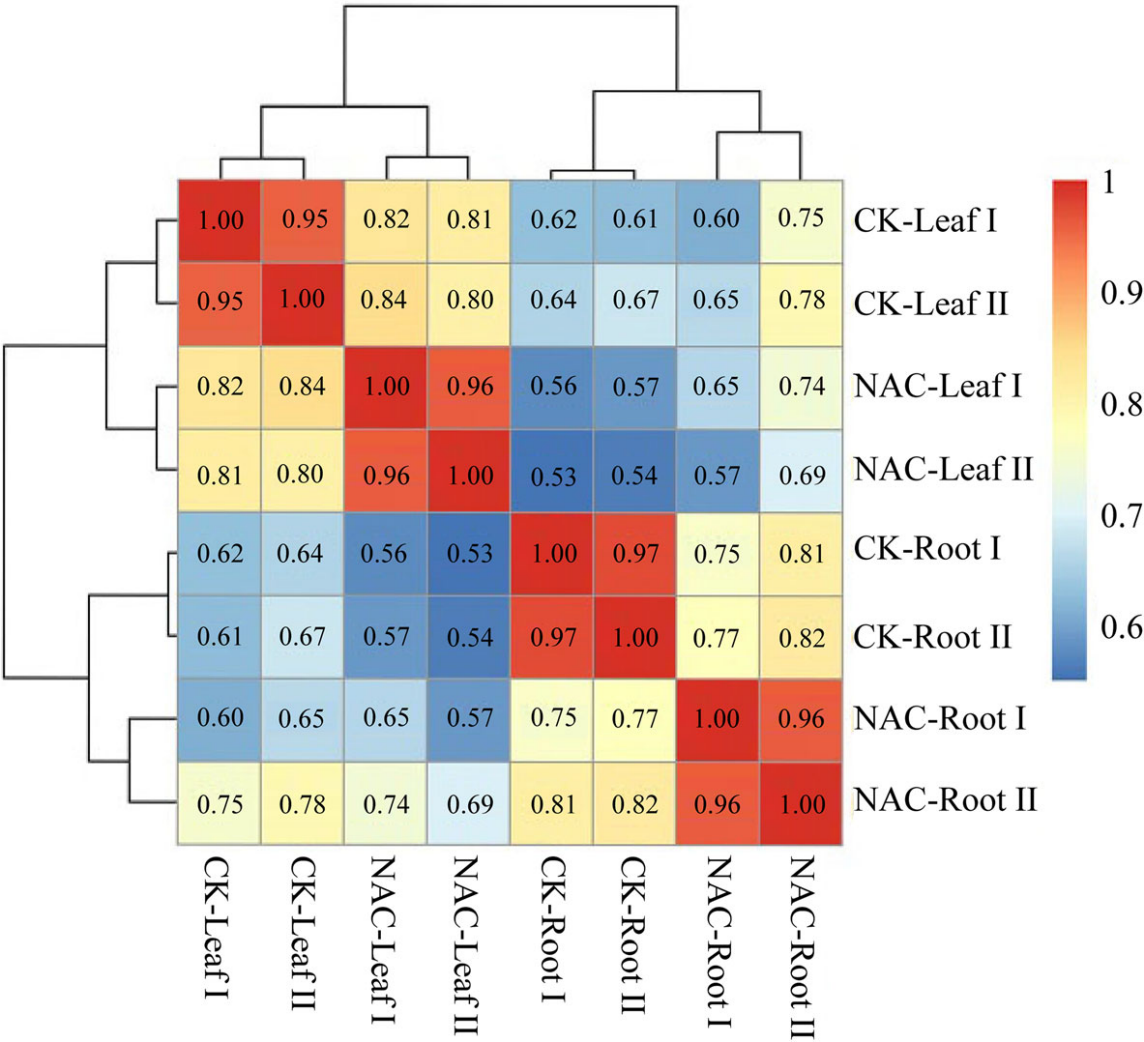
Correlation analysis of the samples used for sequencing. The sample numbers are indicated, and the values in the squares are the Pearson correlation coefficients calculated by R Studio

Two biological replicates were set up for each treatment. For each treatment, 10 roots and 10 leaves from 10-day-old seedlings were collected separately and used to prepare cDNA libraries. The sequencing results showed that the correlation between the biological replicates was high, indicating that the sequencing data were repeatable and reliable (Fig. 4). After joining overlapping reads and removing low-quality sequences from the raw reads, high-quality, clean reads of Q > 20 were retained: 255,820,114 reads for the leaf samples and 283,192,836 reads for the root samples. In total, 461,062,200 filtered clean reads were compared to wheat reference genomes using Tophat2. In total, 452,832,933 reads (85.6%) were mapped to gene regions, 97.7% (442,365,747) of which were mapped to exon regions.

**Fig. 5:**
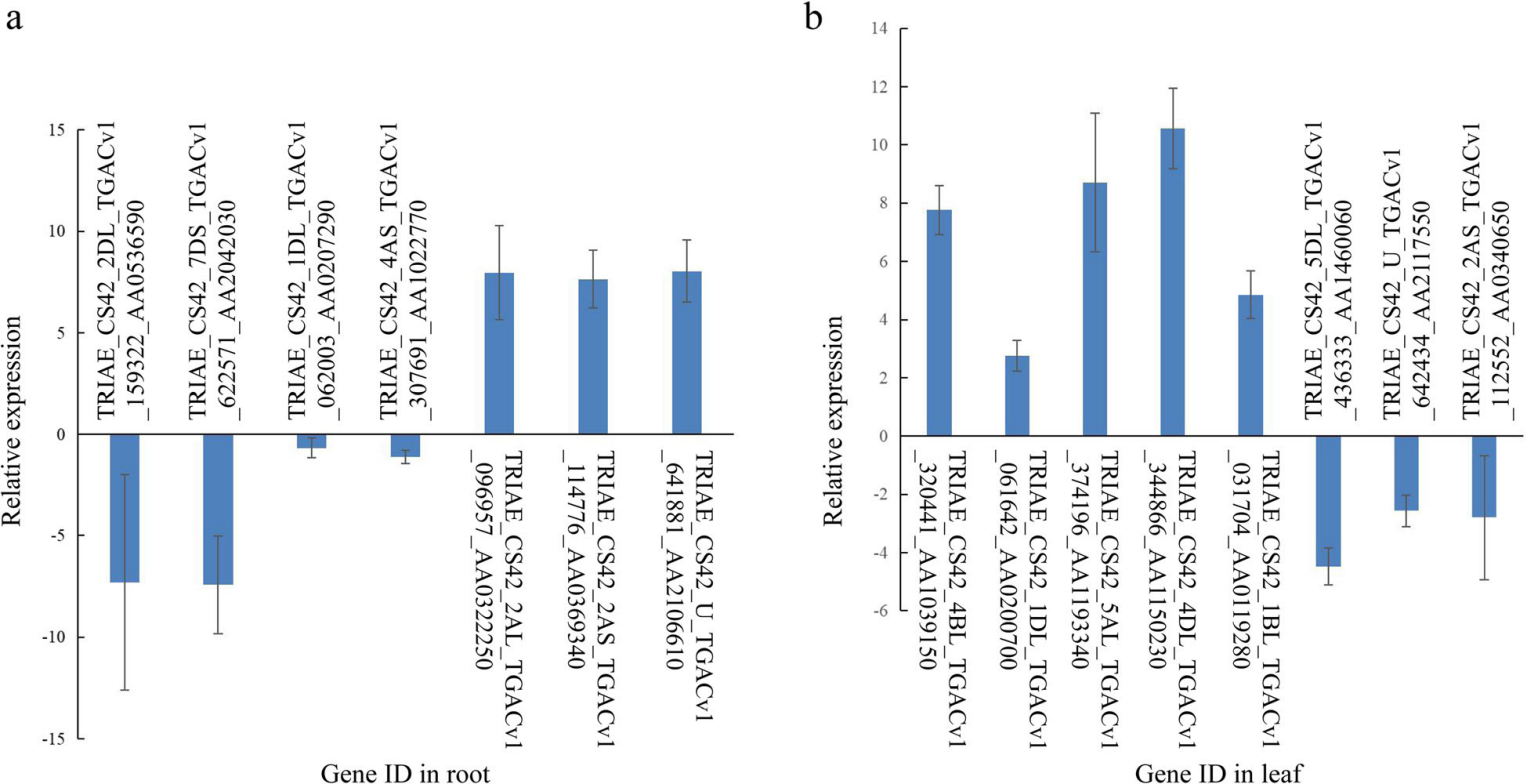
Validation of the RNA sequencing data by RT-qPCR. Comparison of log_2_-transformed relative expression of genes in seedling grown on NAC versus N6

**Fig. 6:**
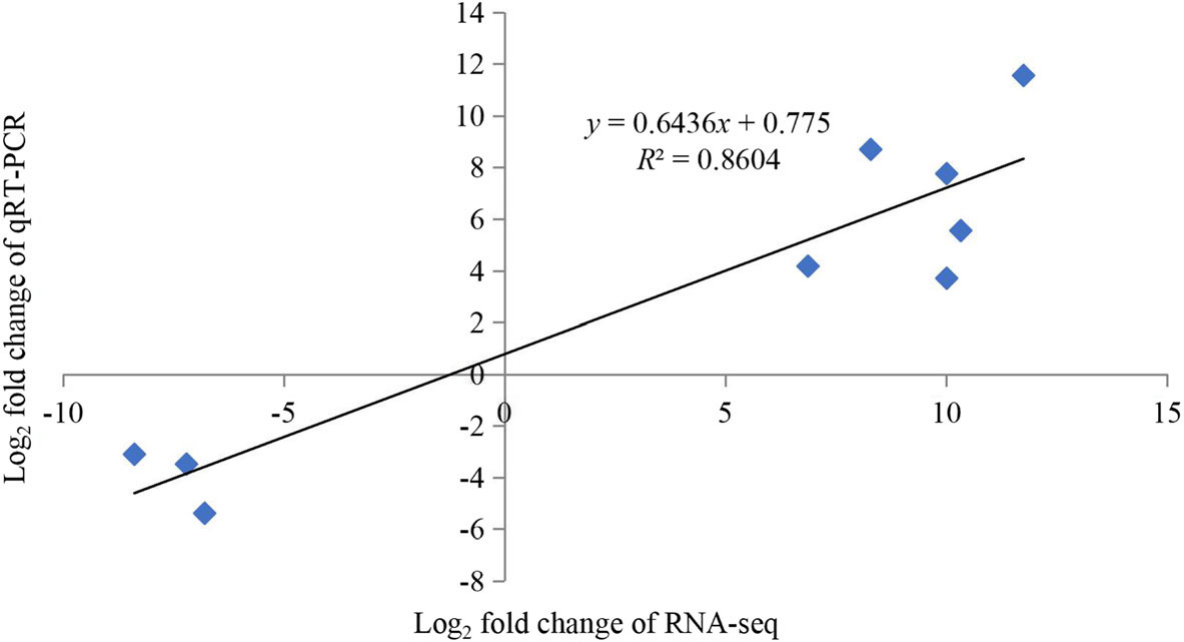
Correlation analysis of DEGs between RNA-seq and qRT-PCR data. The scatter plot indicates the log_2_-transformed gene expression values in RNA-seq and qRT-PCR

To validate the RNA sequencing data, 15 DEGs were randomly selected and assessed by quantitative reverse-transcription PCR (qRT-PCR). Gene expression was determined relative to a control (seedlings grown on N6), which was set as 1.0. The qRT-PCR results showed that the relative expressions of four root genes and three leaf genes were lower in seedlings grown on NAC than those of the seedlings grown in the control. Three root genes and five leaf genes were expressed at significantly higher levels in seedlings grown on NAC than in the control (Fig. 5). Correlation between differential gene expression levels in RNA-seq and qRT-PCR was analyzed after log_2_ transformation. The Pearson correlation coefficient was 0.992, which indicated significant correlation at the 0.01 level. Linear correlation analysis showed that the coefficient of correlation between RNA-seq and qRT-PCR data was 0.643, the *R*^2^ value was 0.860, which is higher than 0.85 (Fig. 6), indicating that RNA-seq and qRT-PCR data were consistent.

### Kyoto encyclopedia of genes and genomes (KEGG) enrichment analysis of DEGs

**Fig. 7:**
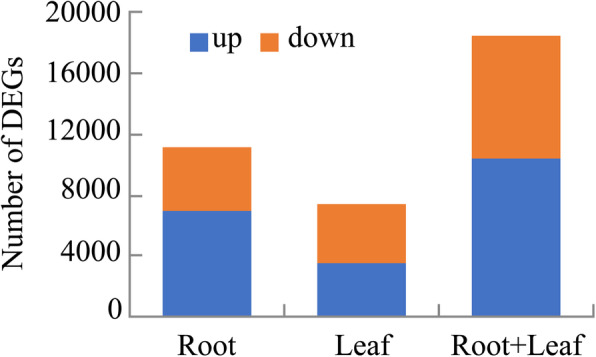
DEGs in wheat roots and leaves from seedlings grown on NAC or N6 (control condition)

**Fig. 8:**
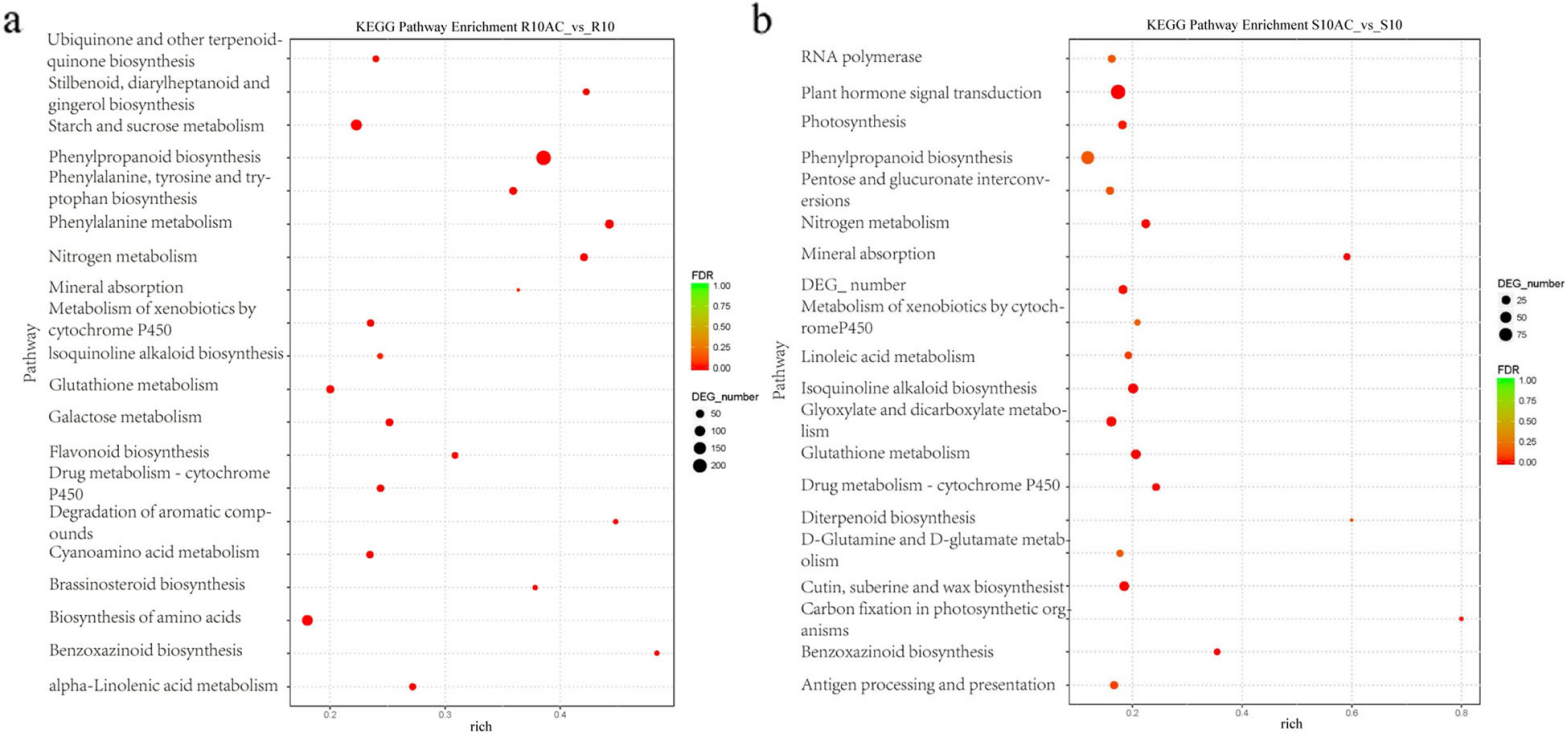
Major pathways differentially regulated by AC in roots and leaves as revealed by KEGG enrichment analysis. KEGG pathways are shown in the ordinate. Dot size indicates how many DEGs are annotated to the pathway. The 20 most significant pathways identified in roots and leaves are shown

**Fig. 9:**
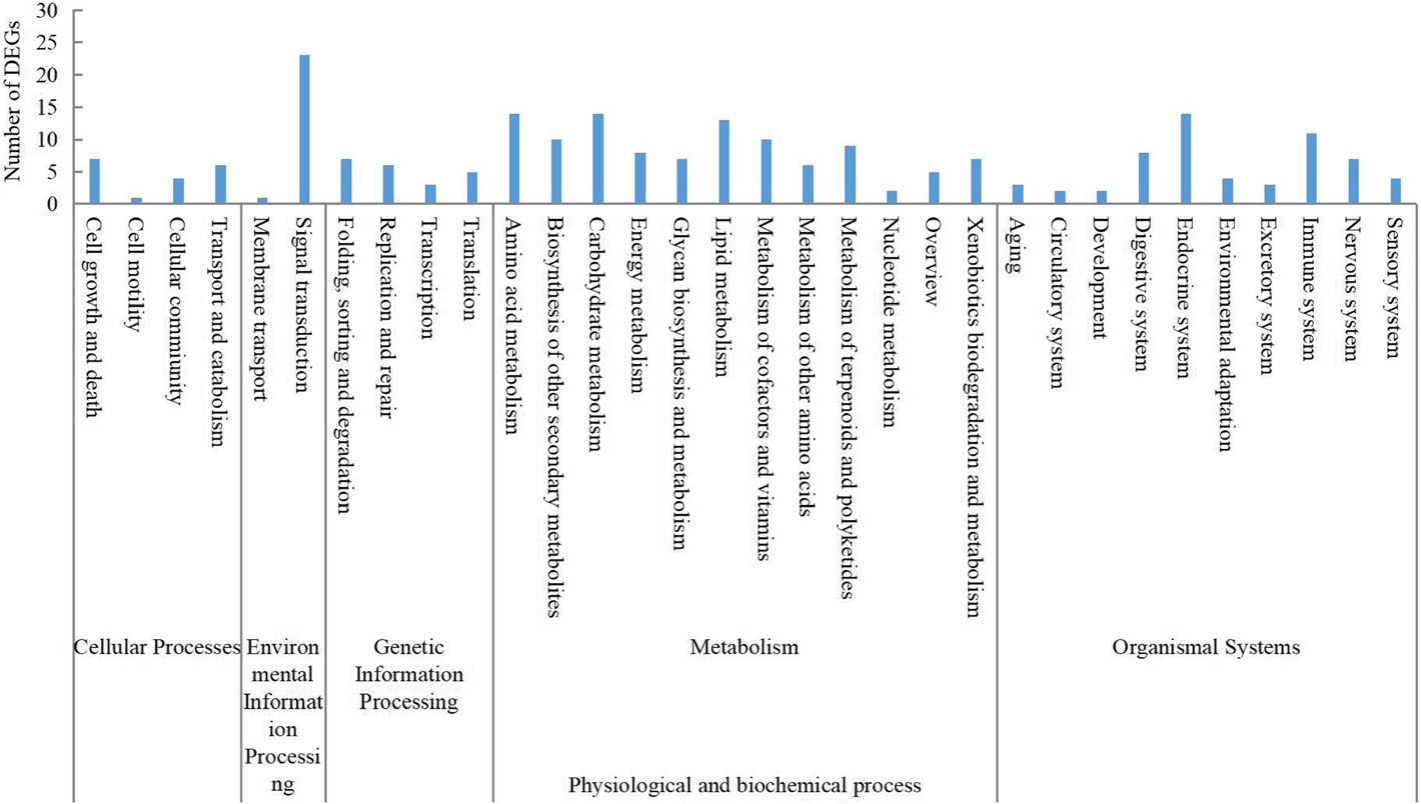
Major pathways differentially regulated by AC in wheat seedings as indicated by KEGG enrichment analysis

In total, 18,555 DEGs were identified using DESeq (version 1.18.0), including 1182 DEGs in the roots and 7373 DEGs in the leaves, and 1612 DEGs in common between the roots and leaves. Among the DEGs, 9460 were upregulated in seedlings grown on NAC compared to N6 medium, and 7483 were downregulated (Fig. 7). To identify the functional pathways the DEGs are involved in, we used KEGG pathway analysis, including 254 KEGG functional pathways. In total, 226 KEGG pathways were commonly differentially regulated by AC in the roots as well as leaves. Among these, “metabolic pathways” (105, 39.10%) represented the largest group, followed by “organismal systems” (58, 25.66%), “environmental information processing” (24, 10.62%), “genetic information processing” (21, 9.29%), and “cellular processes” (18, 7.96%). *P* < 0.05 was considered as a threshold for screening. Further, 37 KEGG pathways were enriched for AC-regulated genes in the roots, and 30 KEGG pathways in the leaves (Fig. 8). In the roots, the three most gene-enriched pathways were “phenylpropanoid biosynthesis”, “starch and sucrose metabolism”, and “biosynthesis of amino acids”. In the leaves, the three most enriched pathways were “plant hormone signal transduction”, “phenylpropanoid biosynthesis”, and “glyoxylate and dicarboxylate metabolism”. By comparison, we found that “phenylpropanoid biosynthesis”, “plant hormone signal transduction”, “starch and sucrose metabolism”, “biosynthesis of amino acids”, and other metabolic pathways were the main gene-enriched pathways in wheat seedlings (Fig. 9). We analyzed three major metabolic pathways, i.e., “phenylpropanoid biosynthesis”, “plant hormone signal transduction”, and “starch and sucrose metabolism” in more detail. In these pathways, there were 29 DEGs between the NAC and N6 groups. Twenty-one of these genes were upregulated, including genes related to cell differentiation, seedling growth, and enhanced stress and disease resistance (e.g., *PLA*, *HCT*, *ZIM*, and *JAC*), and eight of them were downregulated, and were mainly related to the inhibition of plant growth (e.g., *BKI1, ARR-B*, *DELLA*, and *ABF*) (Table 1).

**Table 1:** Three pathways and major related genes differentially expressed in wheat seedlings grown on medium containing CA, as indicated by KEGG enrichment analysis

Pathway	Gene ID	Fold change (NAC/N6)	Expression in NAC	Description
Phenylpropanoid biosynthesis	TRIAE_CS42_1BS_TGACv1_049914_AA0164150	47.15	up	(PAL) phenylalanine ammonia-lyase
	TRIAE_CS42_1DS_TGACv1_080107_AA0239320	12.22	up	(PAL) phenylalanine ammonia-lyase
	TRIAE_CS42_1BS_TGACv1_049965_AA0164870	10.79	up	(PAL) phenylalanine ammonia-lyase
	TRIAE_CS42_2AL_TGACv1_096113_AA0317230	10.21	up	(PAL) phenylalanine ammonia-lyase
	TRIAE_CS42_6DL_TGACv1_527273_AA1701630	8.24	up	(PAL) phenylalanine ammonia-lyase
	TRIAE_CS42_1AS_TGACv1_019041_AA0058710	7.14	up	(PAL) phenylalanine ammonia-lyase
	TRIAE_CS42_3AL_TGACv1_194598_AA0636520	9.78	up	(CYP73A) trans-cinnamate 4-monooxygenase
	TRIAE_CS42_3B_TGACv1_220699_AA0715850	6.61	up	(CYP73A) trans-cinnamate 4-monooxygenase
	TRIAE_CS42_2BS_TGACv1_148390_AA0492590	6.15	up	(CYP73A) trans-cinnamate 4-monooxygenase
	TRIAE_CS42_5AL_TGACv1_378388_AA1253080	5.77	up	(CYP73A) trans-cinnamate 4-monooxygenase
	TRIAE_CS42_3DS_TGACv1_271628_AA0904230	5.50	up	(CYP73A) trans-cinnamate 4-monooxygenase
	TRIAE_CS42_6DS_TGACv1_543204_AA1737020	4.28	up	(COMT) caffeic acid 3-O-methyltransferase
	TRIAE_CS42_6BS_TGACv1_514476_AA1660340	2.19	up	(COMT) caffeic acid 3-O-methyltransferase
	TRIAE_CS42_2BL_TGACv1_132718_AA0439360	7.96	up	(CYP84A, F5H) ferulate-5-hydroxylase
	TRIAE_CS42_2AS_TGACv1_113803_AA0360840	6.15	up	(4CL) 4-coumarate—CoA ligase
	TRIAE_CS42_6BL_TGACv1_502904_AA1626620	5.14	up	(4CL) 4-coumarate—CoA ligase
	TRIAE_CS42_7BS_TGACv1_591841_AA1923260	8.26	up	(HCT) shikimate O-hydroxycinnamoyltransferase
	TRIAE_CS42_2DS_TGACv1_178855_AA0601830	5.80	up	(HCT) shikimate O-hydroxycinnamoyltransferase
	TRIAE_CS42_7AS_TGACv1_569782_AA1824070	5.73	up	(HCT) shikimate O-hydroxycinnamoyltransferase
	TRIAE_CS42_3AL_TGACv1_194329_AA0631150	2.42	up	(CYP98A, C3’H) 5-O-(4-coumaroyl)-D-quinate 3′-monooxygenase
	TRIAE_CS42_7BS_TGACv1_592306_AA1935390	3.59	up	caffeoyl-CoA O-methyltransferase
	TRIAE_CS42_7DS_TGACv1_621454_AA2016210	4.05	up	caffeoyl-CoA O-methyltransferase
	TRIAE_CS42_5DL_TGACv1_436308_AA1459900	6.25	up	(CCR) cinnamoyl-CoA reductase
	TRIAE_CS42_5BL_TGACv1_406204_AA1342180	5.56	up	(CCR) cinnamoyl-CoA reductase
	TRIAE_CS42_5AL_TGACv1_375041_AA1214580	5.15	up	(CCR) cinnamoyl-CoA reductase
Plant hormone signal transduction	TRIAE_CS42_4BL_TGACv1_321177_AA1056660	2.09	up	(ARR-A) two-component response regulator ARR-A family
	TRIAE_CS42_3B_TGACv1_221378_AA0738750	20.04	up	(PYL) abscisic acid receptor PYR/PYL family
	TRIAE_CS42_7DL_TGACv1_602538_AA1959790	2.44	up	(EBF1_2) EIN6-binding F-box protein
	TRIAE_CS42_6BS_TGACv1_514535_AA1661150	2.40	up	(EBF1_2) EIN3-binding F-box protein
	TRIAE_CS42_3DL_TGACv1_251912_AA0885890	3.87	up	(EIN3) ethylene-insensitive protein 3
	TRIAE_CS42_2DS_TGACv1_178626_AA0598480	2.61	up	(BZR1_2) brassinosteroid resistant ½
	TRIAE_CS42_1BL_TGACv1_030488_AA0092220	5.34	up	(JAR1_4_6) jasmonic acid-amino synthetase
	TRIAE_CS42_4BL_TGACv1_320580_AA1043710	6.24	up	(JAZ) jasmonate ZIM domain-containing protein
	TRIAE_CS42_5BL_TGACv1_405157_AA1321310	24.45	up	(PR1) pathogenesis-related protein 1
	TRIAE_CS42_7DS_TGACv1_625472_AA2065280	8.73	up	(PR1) pathogenesis-related protein 1
	TRIAE_CS42_3B_TGACv1_221831_AA0750870	0.32	down	(AHP) histidine-containing phosphotransfer protein
	TRIAE_CS42_7AS_TGACv1_569714_AA1822400	0.31	down	(ARR-B) two-component response regulator ARR-B family
	TRIAE_CS42_7AS_TGACv1_569714_AA1822400	0.31	down	(DELLA) DELLA protein
	TRIAE_CS42_3AL_TGACv1_197036_AA0664480	0.19	down	(ABF) ABA responsive element binding factor
	TRIAE_CS42_5BL_TGACv1_404247_AA1292100	0.22	down	(BKI1) BRI1 kinase inhibitor 1
	TRIAE_CS42_3DL_TGACv1_250531_AA0869810	0.48	down	(NPR1) regulatory protein NPR1
Starch and sucrose metabolism	TRIAE_CS42_2AS_TGACv1_114089_AA0363940	10.52	up	(otsB) trehalose 7-phosphate phosphatase
	TRIAE_CS42_2DS_TGACv1_178535_AA0597240	4.84	up	(otsB) trehalose 8-phosphate phosphatase
	TRIAE_CS42_1AL_TGACv1_003899_AA0051890	2.29	up	(TREH, treA, treF) alpha, alpha-trehalase
	TRIAE_CS42_1DL_TGACv1_061138_AA0186610	2.03	up	(TREH, treA, treF) alpha, alpha-trehalase
	TRIAE_CS42_3DL_TGACv1_249164_AA0840030	2.79	up	(scrK) fructokinase
	TRIAE_CS42_7DS_TGACv1_624145_AA2059200	Inf	up	(glgA) starch synthase
	TRIAE_CS42_4DS_TGACv1_361541_AA1169860	0.31	down	sucrose-phosphate synthase
	TRIAE_CS42_6DL_TGACv1_526359_AA1680390	0.08	down	(AMY, amyA, malS) alpha-amylase
	TRIAE_CS42_2DL_TGACv1_158310_AA0515330	0.43	down	(GBE1, glgB) 1,5-alpha-glucan branching enzyme

## Discussion

### AC stimulates phenylpropane metabolism

The phenylpropane metabolic pathway is of high physiological significance in plants, as it directly and indirectly generates all substances in the phenylpropane skeleton [12]. Nine classes of genes were upregulated in seedlings grown in the presence of AC in the medium, including *PAL*, *CYP73A*, *COMT*, *CYP84A*, and *4CL*. The phenylalanine ammonia-lyase (*PAL*) gene family was actively expressed. PAL catalyzes the nonoxidative deamination of L-phenylalanine to form trans-cinnamic acid and a free ammonium ion [13]. The conversion of the amino acid phenylalanine to trans-cinnamic acid is the entry step for the channeling of carbon from primary metabolism into phenylpropanoid secondary metabolism in plants. The phenylpropane pathway can produce intermediate products such as trans-cinnamic acid, coumaric acid, ferulic acid, and sinapic acid. These intermediate products can be converted into coumarin, chlorogenic acid, and trans-coumaric coenzyme A ester, which can be further converted into secondary metabolites such as lignin, flavonoids, isoflavones, alkaloids, and benzoate glycosides. These products play vital roles in plant growth and development, and the contents of these substances are closely related to PAL activity, which is of great importance in plant physiology [14, 15]. One of the physiological roles of PAL is to promote cell differentiation and plant growth [16]. This study revealed that addition of AC to the growth medium can accelerate seedling growth, at least in part, by promoting *PAL* expression.

### AC affects plant hormone signal transduction

Using KEGG enrichment analysis, 169 DEGs were mapped to plant hormone signal transduction pathways, which represented the second largest group among the mapped functional pathways. Ninety-six DEGs mapped to this pathway were upregulated, and 73 DEGs were downregulated in the NAC compared to the N6 group. Addition of AC to the seedling culture medium increased the expression of *EIN3*, *BZR1*, *JAR1*, *JAZ*, and *PR1*. These genes are known to be involved in plant hormone signal transduction pathways, which directly or indirectly play an important role in regulating stress resistance or disease resistance [17–21]. For example, PR1 is a water-soluble protein that is produced by plants in response to infection by pathogens or stimulation by biotic factors. Its main functions include attacking pathogens, degrading cell wall macromolecules, degrading pathogen toxins, and binding viral coat protein to plant receptor molecules [22]. Inversely, the expression of genes involved in the regulation of plant growth inhibition (*BKI1*, *ARR-B*, *DELLA*, and *ABF*) was reduced (Table 1). For example, DELLA proteins are transcription factors that negatively regulate gibberellin signaling [23]. Our study showed that the addition of AC to the culture medium stimulated the expression of plant hormone signaling-related genes involved in resistance in wheat seedlings.

## Conclusions

AC can significantly promote wheat seedling growth, and this study revealed it likely did so, at least in part, by promoting the expression of certain genes in the phenylpropanoid biosynthesis pathway related to cell differentiation and seedling growth and that of hormone signal transduction-related genes involved in resistance. Our transcriptome data provide new insights into gene expression influenced by AC. AC stimulated gene expression related to phenylpropanoid biosynthesis to promote cell differentiation and seedling growth as well as gene expression related to stress and disease resistance, and suppressed the expression of growth-inhibiting genes through the regulation of plant hormone signaling. Results of this study preliminarily show that AC can significantly promote the molecular mechanisms underlying wheat seedling growth, which will be helpful for further studies on wheat growth.

## Methods

### Plant materials and growth conditions

Winter wheat Liangxing 99 (*Triticum aestivum)* from Dezhou liangxing seed research institute, a popular cultivar cultivated in the Huang-huai winter wheat region of China, was used. In May 2016, a young ear at 15 days post blooming was adopted in the field. The middle part of young spikes of wheat was peeled, sterilized with 1.5% NaClO for 15 min, and rinsed thoroughly with distilled water. Then, immature embryos were peeled off and the scutella were inoculated downward in base medium (N6 supplemented with 0.02 mg/L NAA and 0.05 mg/L 6-BA) or NAC (base medium supplemented with 4 g/L AC). Ten biological replicates were prepared for each group, with 10 immature embryos in each replicate. Ten 5-day-old and 10 10-day-old seedlings were taken to determine dry weight, leaf and root fresh weights, leaf length, leaf number, first-leaf area, root length, and root number. Biochemical indices related to growth were measured in 10-day-old seedlings. Root activity was determined by naphthylamine TCC colorimetry [24]. Soluble sugars were determined by anthrone colorimetry [25], soluble protein content was determined by Coomassie bright blue G-250 staining [26], and total phenol was determined by the tannin method [27]. Trait differences were analyzed by statistical analysis using SPSS 18.0 software (IBM, USA).

From 40 10-day-cultured seedlings grown on N6 and NAC media, roots and leaves were collected separately. Each sample comprised 20 independent leaves or 20 independent roots; two biological replicates were paired for each sample, immediately frozen in liquid nitrogen, and stored at − 80 °C.

### RNA isolation and cDNA library construction and sequencing

Total RNA was isolated using a TRIzol total RNA extraction kit (Invitrogen, USA), which yielded ~ 10 μg of total RNA per sample. RNA quality was examined by 0.8% agarose gel electrophoresis and spectrophotometry. High-quality RNA with 28S:18S > 1.5 and a 260/280 absorbance ratio of 1.8–2.2 was used for library construction and sequencing. Illumina HiSeq library construction was performed according to the manufacturer’s instructions (Illumina, USA). Magnetic beads with poly-T oligos attached were used to purify mRNA from total RNA. mRNA was broken into 200–300 bp fragments using ion interruption. Using mRNA as the template, 6-base random primers and reverse transcriptase were used to synthesize the first cDNA chain, which was used as a template for the synthesis of the second chain of cDNA, where the base T was replaced with the base U. After library construction, library fragments were enriched by PCR amplification and selected according to a fragment size of 300–400 bp. The library was quality-assessed using an Agilent 2100 Bioanalyzer (Agilent, USA). The library was sequenced using the Illumina HiSeq sequencing platform, using paired-end sequencing to generate raw reads (Shanghai Personal Biotechnology Co., Ltd., China).

### RNA-sequencing data analysis

Raw reads were filtered before data analysis; high-quality reads with Q > 20 were retained for subsequent analysis. Reference genome data were collected from the Ensembl database (http://www.ensembl.org/). The reference genome index was created using Bowtie2 software [28]. The reads were filtered by Tophat2 (http://tophat.cbcb.umd.edu/) and compared to the reference index The read count for each gene was determined using HTSeq0.6.1p2 (https://github.com/genepattern/HTSeq.Count) as the original gene expression level. Expression levels were normalized using reads per kilo bases per million reads (RPKM), with RPKM values > 1 considered as the gene expression standard [29]. Differential gene expression was determined using DESeq, and genes with a more than a two-fold change in expression (log_2_ fold change > 1) and *P* < 0.05 were considered as DEGs [30]. KEGG pathway analysis was used to analyze the metabolic pathways and signaling pathways the DEGs were primarily involved in.

### RT-qPCR analysis

To validate the DEGs identified by RNA sequencing, 15 candidate DEGs were randomly selected for RT-qPCR analysis. The gene names and primer information are listed in Table 2. The wheat housekeeping gene, *TaRP15*, was used as an internal control for normalization [31]. Three biological replicates were paired for each sample. cDNA was transcribed from 1 μg RNA using a PrimeScript™ RT reagent Kit with gDNA Eraser (TakaRa, Japan). qPCRs were run using a SYBR Premix Ex Taq kit (TakaRa) in an ABI ViiATM7 instrument (Applied Biosystems, USA). The 2^–ΔΔCT^ method was used to quantify relative target gene expression [32].

**Table 2:** Primers used for qRT-PCR

Gene ID	Forward (5′ → 3′)	Reverse (5′ → 3′)
TaRP15	GCACACGTGCTTTGCAGATAAG	GCCCTCAAGCTCAACCATAACT
TRIAE_CS42_2DL_TGACv1_159322_AA0536590	CCCTGGGAGACTTACGATGGA	CCCGTGCTTGCTCTTGTGGAT
TRIAE_CS42_7DS_TGACv1_622571_AA2042030	GCCAACCGCGTGGACGAGTA	CCATCCCTGCCGTATGACCT
TRIAE_CS42_1DL_TGACv1_062003_AA0207290	TGTTCCACATCGGTGACTTCTTC	CCCGCTGATTGGGTTTGC
TRIAE_CS42_4AS_TGACv1_307691_AA1022770	ATACGGGTTCATATCCTTACCG	CCCAGCCACGCTTCACA
TRIAE_CS42_2AL_TGACv1_096957_AA0322250	AGGTGAACAACGGCAAGGTG	AGGATGAGGTCGCTGATTGG
TRIAE_CS42_2AS_TGACv1_114776_AA0369340	GGATGCCCTGGTCCAGAAGA	AGGTGGTCGAGCGGGTTGTC
TRIAE_CS42_U_TGACv1_641881_AA2106610	GGACGGGAACTTCATCGC	TGGTCGGAGTAGGTCGTACATT
TRIAE_CS42_4BL_TGACv1_320441_AA1039150	CCTCCGCTCTGCCAATA	CCAATACGATCTGCCACC
TRIAE_CS42_1DL_TGACv1_061642_AA0200700	AAGTCGTGGATAGTGCCTGGAT	GGTTGCTGGGTCCGTTGA
TRIAE_CS42_5AL_TGACv1_374196_AA1193340	TGAACTCCGTCATCATCGCACAG	CGGCGTTGGCAAACTCCTCT
TRIAE_CS42_4DL_TGACv1_344866_AA1150230	CCCTTGTAACTCCTTCCTC	TTCATAGTCGCCATCACC
TRIAE_CS42_1BL_TGACv1_031704_AA0119280	TTCAACAAGCTGGAGGTTCG	GCCAAATGCTCGTAGGAGTAAA
TRIAE_CS42_5DL_TGACv1_436333_AA1460060	GTGACCGTGGACGAAGTGAT	GCTGTTGGTGATGCGAAAGT
TRIAE_CS42_U_TGACv1_642434_AA2117550	TGGAACACCGACGACCGC	CTGCTCGCTGGAGAAGCTGAC
TRIAE_CS42_2AS_TGACv1_112552_AA0340650	ATGAGGCAAGTATGGAGAACA	GCAATGAGCCGAGTAATAGAA

## Data Availability

Supplementary data to this article can be found online at https://www.ncbi.nlm.nih.gov/sra/PRJN556084.

## Abbreviations

AC: Activated charcoal
DEGS: Differentially expressed genes
KEGG: Kyoto Encyclopedia of Genes and Genomes
NAC: Base medium with 4 g/L AC
PAL: Phenylalanine ammonia-lyase
qRT-PCR: Quantitative real-time polymerase chain reaction
*4CL*: 4-Coumarate--CoA ligase gene
*ARR-B*: Two-component response regulator ARR-B family gene
*BKI1*: BRI1 kinase inhibitor 1 gene
*BZR1*: Brassinosteroid resistant 1/2 gene
*COMT*: Caffeic acid 3-O-methyltransferase gene
*CYP73A*: Trans-cinnamate 4-monooxygenase gene
*EIN3*: Ethylene-insensitive protein 3 gene
*JAR1*: Jasmonic acid-amino synthetase gene
*JAZ*: Jasmonate ZIM domain-containing protein gene
N6: N6 base medium (supplemented with 0.02 mg/L NAA, 0.05 mg/L 6-BA)
*PLA*: Phenylalanine ammonia-lyase gene
*PR1*: Pathogenesis-related protein 1 gene
